# Evaluation of a new concept to improve and organize clinical practice in nursing education: a pilot-study

**DOI:** 10.1186/s12912-024-01888-y

**Published:** 2024-03-26

**Authors:** Helle Skou Thomsen, Britt Egeris Jørgensen, Jette Kynde Schøtz, Line Muff Bech, Lea Ladegaard Grønkjær

**Affiliations:** 1 Department of Education, Department of Gastroenterology, University Hospital of Southern Denmark, Esbjerg, Denmark; 2 Nursing Education, University College South Denmark, Esbjerg, Denmark; 3 Department of Gastroenterology, University Hospital of Southern Denmark, Finsensgade 35, 6700 Esbjerg, Denmark

**Keywords:** Clinical practice, Learning environment, Nursing education, Nursing students

## Abstract

**Background:**

Nursing students may experience clinical practice as unsafe due to the interactions with patients, fear of making mistakes, lack of clinical experience and supervision, which results in anxiety and stress. Thus, interventions to improve and organize the learning environment in clinical practice for nursing students are warranted, and the aim of this pilot-study was to evaluate a new concept of clinical practice in order to get insight on the different initiatives and gain knowledge for further developing.

**Methods:**

The new concept consisted of nursing students being affiliated to the same department during their clinical practices, reflective supervision, and participation in a self-compassion course. Data was collected using questionnaires and focus group interviews of 17 nursing students, 17 clinical supervisors, and 14 head nurses. A mixed-methods strategy was employed to give the study a pragmatic approach. Finding from the questionnaires and focus group interviews were analyzed separately and then weaved together into themes.

**Results:**

The results generated four themes: Information and involvement before and during the new concept, Learning outcomes, safety, and well-being, Impact of reflective supervision and self-compassion course, and Transition from study life to working life. In general, the participating nursing students, clinical supervisors, and head nurses had positives experiences regarding the new concept. They felt well-informed, and they experienced that it contributed to a safe learning environment, increased well-being, strengthened the relationship between nursing students and clinical supervisors and healthcare staff at the department, and prepared the nursing students to working life.

**Conclusion:**

Our results complement the suggestion that improved quality of clinical practice for nursing students is an effective strategy to establish a safe and supportive learning environment that contribute with satisfaction, successful experiences, and attraction of future nurses. However, further intervention studies are needed to compare the effect of the new concept with traditional clinical practice.

**Supplementary Information:**

The online version contains supplementary material available at 10.1186/s12912-024-01888-y.

## Introduction

Nurses are vital to the healthcare system and consist of the largest part of healthcare professionals. However, the nursing profession worldwide faces shortages due to the increase in the ageing population and people living with chronic diseases, inequitable workforce distribution with more nurses retiring, high turnover, and a high number of student dropouts at the nursing education [1, 2].

Dropout from nursing education is problematic considering the severe shortage of nurses, and it may have severe consequences for the healthcare system. Reasons for dropout include financial problems, personal reasons, but also the educational structure of the nursing education such as theory–practice gap and unpleasant experiences and lack of support during clinical practice. Clinical practice refers to the clinical workplace for example hospitals in which nursing students complete their clinical placements as part of their education [3, 4].

Clinical practice is of high importance in nursing education as it is in this context that nursing students acquire clinical skills, apply theoretical knowledge to practice, and develop clinical reasoning and problem-solving skills [5]. In addition, studies have indicated that the experience of clinical practice influence nursing students learning outcome and, especially in the last part of the education, also facilitate the transition from study to working life, strengths nursing students commitment to the nursing profession, and is important in relation to the choice of future workplace [6–8]. However, several studies have shown that nursing students experience clinical practice as unsafe due to difficulties in the interactions with patients, fear of making mistakes, lack of clinical experience and supervision, which may result in anxiety and stress [9–11]. Thus, it appears that there is a need for interventions to improve and organize clinical practice for nursing students as an potentially effective strategy to establish a safe and supportive learning environment that contribute with satisfaction, successful experiences, and attraction of future nurses [11]. With this background, the aim of this pilot-study was to evaluate a new concept of clinical practice in order to get insight on the different initiatives and gain knowledge for further developing of the concept.

## Methods

This was a pilot-study to evaluate a new concept to improve and organize clinical practice for nursing students at University Hospital of Southern Denmark, Esbjerg, and the results are reported according to the CONSORT extension to pilot and feasibility trials [12, 13].

### Setting of the study

The nursing education in Denmark is 3½ years consisting of seven semesters (210 European Credit Transfer System (ECTS) points). Clinical practice are included in all semester and make up 90 ECTS points of the education. Nursing students from the different semesters do a large part of their clinical practice in different departments at the hospitals. In addition, practice is also carried out in primary health care centers, all of them belonging to the same healthcare region.

**Fig. 1:**
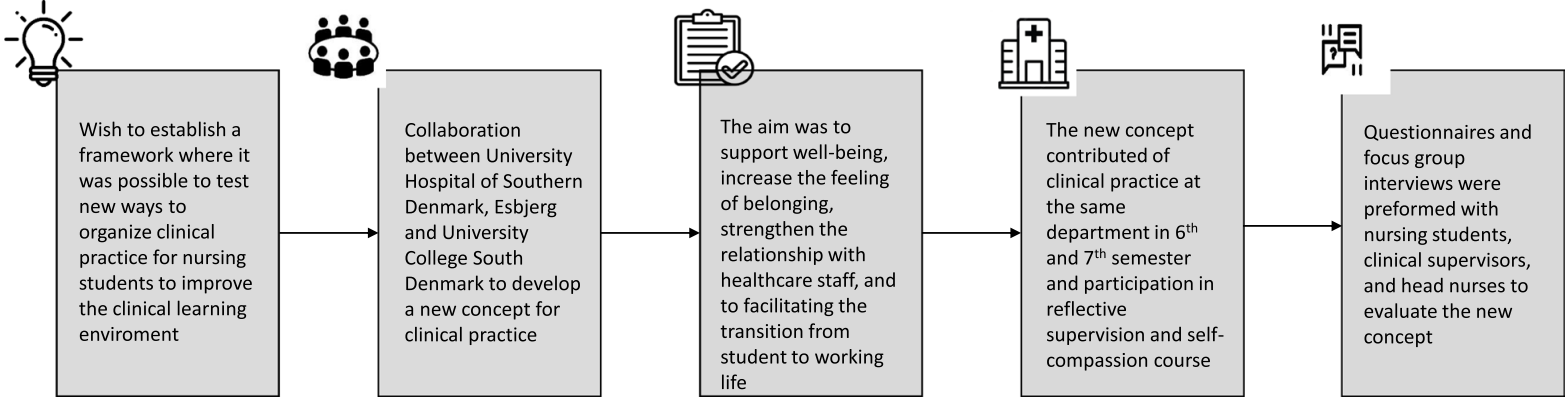
Overview of the new concept for clinical practice

University Hospital of Southern Denmark, Esbjerg and University College South Denmark have collaborated on developing a new concept of clinical practice to establish a framework for a safe learning environment where it is possible to test new ways of improving and organizing clinical practice. In connection with this concept, nursing students are affiliated at the same department at their clinical practice in both 6th and 7th semester. The aim was to create a clinical learning environment that could support the well-being of the nursing students, increase the feeling of belonging and strengthen the relationship with the healthcare staff at the department. In addition, the intension was to give nursing students confidence to become more independent, which could contribute to facilitating the transition from student to working life. The concept also included initiatives such as reflective supervision during the clinical practice of every semester together with fellow nursing students, a clinical supervisor from the University Hospital of Southern Denmark, Esbjerg, and a nurse educator from University College South Denmark, and participation in a self-compassion course for one hour in six weeks. Figure 1 illustrates the new concept.

### Participants

The new concept was tested at 14 of the 37 departments at University Hospital of Southern Denmark, Esbjerg in February 2023. Thus, the pilot-study included 17 nursing students, 17 clinical supervisors, and 14 head nurses, which were all affiliated to these departments. The choice of departments to test the new concept was random, but aimed to represent both general and specialty wards and outpatient clinics. Nursing students were affiliated to the department using random allocation technique.

### Data collection

Data was collected using questionnaires and focus group interviews. Three questionnaires were developed by the authors for this pilot-study. The questionnaires consisted of 15 questions to the nursing students, 13 questions to the clinical supervisors, and 3 questions to the head nurses to evaluate on the new concept for clinical practice (see Supplement Table 1). The questionnaires contained questions on the assessment of the new concept in general (nursing students and clinical supervisors), experience of learning outcomes, feeling of well-being (nursing students, clinical supervisors, and head nurses), experience of reflective supervision, the self-compassion course (nursing students and clinical supervisors), and transition from student to working life (nursing students, clinical supervisors, and head nurses). Despite one question regarding participation in an information meeting, the questions were rated using a five point Likert-scale ranging from: to a very low degree, to a low degree, to some extent, to a high degree, and to a very high degree. In all questionnaires, it was possible to write additional comments. The questionnaires were tested for face validity by one nursing student and one educational consultant to determine whether the questions were clear and understandable. Based on the test, only minor linguistic changes were made.

Afterwards, three focus group interviews were carried out with 15 nursing students divided into two groups, and 12 clinical supervisors. Semistructured interview guides were designed for this pilot-study to capture nursing students and clinical supervisors experience of the new concept for clinical practice including the reflective supervision and the self-compassion course with particular focus on elaborating answers from the questionnaires and explore what was good and what could be improved about the new concept (see Supplement Table 2). The focus group interviews lasted one hour, and were audio and transcribed verbatim. The questionnaire and focus group interviews were carried out by educational consultants (2th, 3th, and 4th author) from University Hospital of Southern Denmark, Esbjerg. The data collection was conducted in May 2023.

### Data analysis

**Fig. 2:**
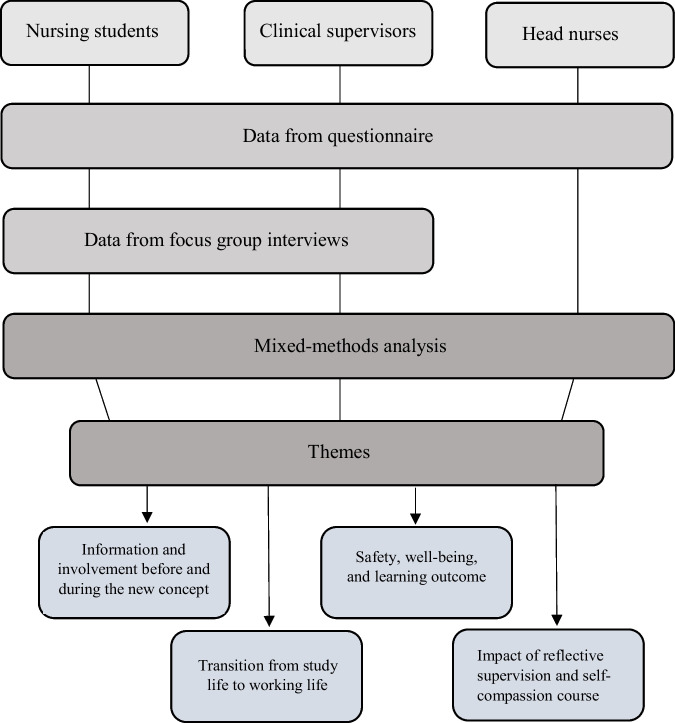
Data collection and data analysis process

A mixed-methods strategy was employed to give the study a pragmatic approach [14]. Figure 2 illustrates the data collection and data analysis process. Data from the questionnaires were analyzed using descriptive statistic and were expressed as numbers and percentages, and the statistical analyses were performed by the computer software program, Microsoft Office Excel 2016.

Comments from the questionnaire were analyzed with inspiration from the Graneheim and Lundman content analysis method, like the focus group interviews [15]. To support reliable and valid inferences, content analysis involves a set of systematic and transparent procedures for processing data [16]. This consist of a stepwise process beginning with several read troughs by the first and last author to obtain an overview of the transcriptions.

Then, the transcriptions were systematically reviewed line by line to identify patterns (i.e. “meaning units”) that expressed segments or quotations that contained information or meaning related to the study aim. Meaning units dealing with the same content were condensed, coded, and organized into categories according to their similarity. The next step involved comparing similarities and differences to reach consensus on the final identified categories. To enhance validity and limit the risk of predetermined interpretation, the first and last author, not having taken part of the data collection earlier, independently performed the analysis. Their preunderstanding consisted of their previous experience as nursing students and present experience as research nurses and knowledge in the field of nursing education from the literature. The analysis and categories were refined and discussed among the whole author group. This made it possible to determine if any preunderstanding could have affected the analysis. The transcripted interviews were reread by the first and last author to ensure that the categories covered all aspect of the data. Representative quotes were chosen and translated into English. Due to the mixed-methods approach, data integration was then performed using a weaving approach. This meant that findings from the questionnaires and focus group interviews were written together on a theme-by-theme basis to present a detailed summary of the results [14].

## Results

Data from the questionnaires and focus group interviews resulted in four themes: *Information and involvement before and during the new concept*, *Learning outcomes, safety, and well-being, Impact of reflective supervision and self-compassion course*, and *Transition from study life to working life*. Answers from the questionnaires are presented in Table 1.

**Table 1:** Answers from the questionnaires

**Nursing students (*N* = 17)**	
Themes	High/very high degree of satisfaction
**Information and involvement during the new concept**	
- Participated in the information meetings	83%
- Felt well informed prior clinical practice	85%
- Felt well informed during clinical practice	53%
- Online information forum kept you informed	76%
**Safety, well-being, and learning outcome**	
- The new concept created a safe learning environment	100%
- Felt being a part of the healthcare staff	82%
- Felt safe knowing you was affiliated to the same department	95%
**Transition from student life to working life**	
- The new concept contributed to prepare you to become a nurse	65%
**Impact of reflective supervision and self-compassion course**	
- Participated in the reflective supervision	100%
- Reflective supervision improving your ability to apply theoretical knowledge to practice	93%
- Reflective supervision brought new perspectives on clinical issues	94%
- Reflective supervision brought new perspectives on development of quality	93%
- Reflective supervision brought new perspectives on interdisciplinary and cross-sectoral collaboration	94%
- Reflective supervision contributed to community of practice	60%
- Self-compassion course improved well-being	30%
**Clinical supervisors (*N* = 17)**	
Themes	High/very high degree of satisfaction
**Information and involvement during the new concept**	
- Participated in at least one information meetings	100%
- Felt well informed	100%
- Online information forum kept you informed	36%
- Felt involved in the new concept	63%
**Safety, well-being, and learning outcome**	
- The new concept improved learning for the nursing students	100%
- The new concept improved well-bring for the nursing students	100%
- It promoted the well-being of the nursing students to be affiliated to the same department	100%
**Transition from student life to working life**	
- The new concept contributed to a higher degree of professional identity	100%
**Impact of reflective supervision and self-compassion course**	
- The reflective supervision improving the nursing students ability to apply theoretical knowledge to practice	72%
- Experienced that the nursing students used elements from the self-compassion course	27%
- The self-compassion course contributed to the nursing students learning and well-being	64%
- The self-compassion course was valuable in regard to your position as a clinical supervisor	72%
- Did you use compassion during your work as a clinical supervisor	45%
**Head nurses (*N* = 17)**	
Themes	High/very high degree of satisfaction
**Safety, well-being, and learning outcome**	
- The new concept improved learning for the nursing students	90%
- The new concept improved well-being for the nursing students	90%
**Transition from student life to working life**	
- The new concept contributed to higher degree of professional identity	100%

### Information and involvement before and during the new concept

As an introduction to the new concept, two information meetings were held for the nursing students, and almost all participated (83%). Nearly all of the nursing students felt that they to a high or very high degree were informed about the new concept before the start of their clinical practice (85%). Likewise did 76% state that an online information forum kept them informed about the initiatives of the new concept during their clinical practice.

All clinical supervisors had participated in at least one information meeting, and all felt informed about the new concept. However, only 36% reported (to a high or very high degree) to be informed using the online information forum during the clinical practice. Of the clinical supervisors, 63% felt involved in the new concept.

### Learning outcomes, safety and well-being

The majority of the nursing students experiences that the affiliation to the same department during clinical practice in 6th and 7th semester contributed to a safe learning environment (80%). Also, 82% felt a high or very high degree of being part of the healthcare staff at the department due to the new concept. Almost all nursing students experiences a high sense of safety, knowing that they were coming back to the same department (95%). However, a concern was raised about the affiliation to the same department in both clinical practices if the nursing students were not interested in the department´s specialty or if the relationship between the nursing student and the clinical supervisor was problematic.

In connection with the focus group interviews with the nursing students, it emerged that they were positive about the collaboration between the hospital and the nursing education. One nursing student reported: “*Nice that the hospital and education are working together. It just does something good and also provides a form of security at the same time. You realize that there are really investing in us*.”

All the clinical supervisors and 90% of the head nurses agree that the affiliation to the same department during clinical practice had a positive effect on the nursing students learning and well-being. During the focus group interview, the clinical supervisors expressed that the new concept allowed time to build a strong relationship between the nursing students and the clinical supervisors. In addition, the clinical supervisors thought that the nursing students felt a greater sense of commitment and responsibility to their department, which also contributed to strengthen the relationship with the healthcare staff. The clinical supervisors also described that the nursing students feeling of safety and well-being due to the new concept contributed to professional development and increased responsibility for own learning.

### Impact of reflective supervision and self-compassion course

All nursing students had participated in reflective supervision, and 93% reported that it to a high or very high degree contributed to improving their ability to apply theoretical knowledge to practice. In addition, the nursing students stated that reflective supervision brought new perspectives on clinical issues (94%), development of quality (93%), and interdisciplinary and cross-sectoral collaboration in clinical practice (94%). One student reported: *“So good to have a bit of theory again because you easily forget when focusing on clinical practice”.*

The focus group interviews revealed that the nursing students preferred small groups during reflective supervision as it contributed to a feeling of safety. In addition, the nursing students described that the reflective supervision created a good learning environment with the possibility of feedback, which also prepared the nursing students for their examination and future nursing practice. One nursing student stated: *“Why have they not done this before. It all makes more sense now. Now we understand”.*

As a suggestion for improvement, the nursing students highlighted a need for more information before attending the reflective supervision so they had the opportunity to be prepared. In addition, a proposal was made to make the reflective supervision online.

Seventy-two percent of the clinical supervisors stated to a high or very high degree that reflective supervision contributed to improve nursing students’ ability to apply theoretical knowledge to practice. In the focus group interviews, the supervisors expressed that the nursing students were excited about the reflective supervision because it contributed to a meaningful learning environment.

All nursing students had participated in the self-compassions course, and 30% stated that the participation to a high or very high degree contributed to their well-being. From the focus group interviews, the nursing students indicated confusion about the purpose of the course and the use in regards to the new concept of clinical practice, and they suggested more information about how to implement self-compassion in practice. This was consistent with the clinical supervisors, they indicated that only 27% of the nursing students to a high or very high degree used elements of the self-compassion course. However, 64% also reported to believe that the compassion approach contributed to the nursing students learning skills and well-being during clinical practice.

### Transition from study life to working life

Sixty-five percent of the nursing students thought to a high or very high degree that the new concept would contribute to prepare them from study to working life. One reported: “*I think it is a good concept and I will be better qualified to be a nurse”.*

All the clinical supervisors and the head nurses agreed to a high or very high degree that the new concept contributed to develop the nursing students professional identity, and thereby readiness to become a nurse.

## Discussion

The aim of this pilot-study was to evaluate a new concept to improve and organize clinical practice in order to get insight on the different initiatives and gain knowledge to further developing of the concept.

In general, the participating nursing students, clinical supervisors, and head nurses had positives experiences regarding the new concept. They felt well-informed about the concept, and they experienced that the affiliation to the same department during clinical practice in 6th and 7th semester contributed to a safe learning environment, increased well-being among nursing students, strengthen relationship with the clinical supervisors and healthcare staff at the department, and prepared the nursing students to working life. In addition, they were satisfied with initiatives such as reflective supervision but to a lower degree the self-compassion course.

Studies have highlighted that successful changes in healthcare organizations requires clear communication and information to allow for preparation and to increase the chances for success [17]. This study showed high degree of information to and involvement of nursing students and clinical supervisors, which may be one of the reasons for the positive evaluation of the new concept. However, more information was wanted in connection with the initiatives reflective supervision and the self-compassion course. In addition, changes such as the new concept of clinical practice are more likely to succeed when the involved healthcare professionals have the opportunity to influence the changes, are prepared for the change, and values the changes [17]. This is in consistent with this pilot-study where the aim is to gain knowledge of the experiences and satisfaction with the concept to further developing.

The results of the evaluation of the pilot-study are consistent with other studies showing that longer clinical practice contributes to the nursing students experience of being part of a workplace, which strengthen the professional recognition and self-confidence, and therefore support the transition from study to working life [10, 18]. In addition, a study has shown that the strengthen relationship between nursing students and healthcare staff enables the staff to push the limits of learning [19]. Concerns about the new concept were raised if the nursing students were not interested in the department´s specialty or if the relationship between the nursing student and the clinical supervisor was problematic. This concern is fair as several studies have shown that a good relationship between nursing students and clinical supervisors is essential for the nursing students learning potential [20, 21].

The nursing students experienced the reflective supervision as positive. They thought it improved their ability to apply theoretical knowledge to practice and brought new perspectives on clinical issues, development of quality, and interdisciplinary and cross-sectoral collaboration in clinical practice, which created a good learning environment and increased their understanding of the nursing practice. This was also underlined by the clinical supervisors. This is in accordance with another study, which show how reflective spaces in clinical practice creates an increased understanding of nursing theory and its relevance for clinical practice, and contributes to achieved professional identity [22]. In addition, a study has highlighted that both situated- and meta learning is necessary in the clinical practice for nursing students to achieve an ultimate learning environment. Situated learning is advice and guidance *in* nursing to develop and improve clinical skills while meta learning is conceived as advice and guidance *about* nursing using theoretical and reflective models to interpret different aspects of a situation and discuss it in professional terms [19].

Studies have shown that nursing students experience clinical practice as unsafe, which may result in anxiety and stress [6–8]. It is importance to talk to the nursing students about their feelings and reactions as they often feel vulnerable, lonely, and lacks support to deal with their emotions. Thus, the nursing students calls for more support to develop their resilience [21, 23]. The challenges of nursing students’ dropout, recruitment and retention of nurses are problematic. Therefore, attention has been made on interventions to emphasize resilience as a positive force for overcoming adversity and improve well-being [24]. In this study, nursing students participated in a self-compassion course. However, only 30% of the nursing students stated that the participation contributed to their well-being, and clinical supervisors indicated that only 27% of the nursing students used elements of the self-compassion course. This is in contrast to other studies who finds positive health benefits such as burnout prevention, improved nursing care, and stress management from attending a self-compassion course [25, 26]. In many of these studies, the effect of self-compassion is measured before and after an intervention using specific self-compassion questionnaires addressing for example burnout, mindfulness, and well-being [27]. In this pilot-study, only one questions concerning to what extent the self-compassion course has contributed to increased well-being was asked. This may be one reason for the difference. In addition, the nursing students indicated confusion about the purpose of the course and the use in regards to the new concept of clinical practice. Thus, another reason may be lack of understanding of the course and how to implement self-compassion in practice. Another study also highlights the need to help cultivate nursing students´ self-compassion not only during clinical practice but also during classroom teaching at the nursing education [28].

A review study has investigated the challenges of supporting nursing students in clinical practice. It found that nursing students faced several challenges in connection with clinical practice such as anxiety and fear, ineffective clinical education, organizational-environmental stress, and socio-cultural challenges. However, most studies only highlighted the presence of such challenges, while few studies dealt with their causes [29]. This pilot-study aimed to evaluate a new concept of clinical practice to create a new clinical learning environment to support nursing students’ independence and well-being, increase the feeling of belonging, strengthen the relationship with the healthcare staff, and facilitate the transition from student to working life. Other studies have also initiated interventions to support nursing student. Thus, a study evaluated initiatives to support the nursing students active learning, engagement, and self-efficacy in clinical practice and found that the *Check-in, Check-out* process engaged the nursing students as active partners in their learning and teaching in their clinical preparation for practice, and helped them to acknowledge and reflect on their accomplishment [30]. In addition, another study found that using a matching program to match the nursing students with clinical workplaces in connection with clinical practice at public health agencies increased the nursing students reflection of own competences and needs as well as developed a successful collaboration between the nursing education and clinical workplaces [31]. Collaboration between nursing educations and clinical workplaces are important for achieving the nursing students learning outcome and facilitate the transition from study to working life. Thus, the higher perceived academic-practice partnership by nursing students, the better interactions with healthcare staff, organizational socialization, and professional self-concept [32]. In this study, the nursing students also described very positive experiences with the collaboration between the hospital and the nursing education.

### Study limitations

The aim of this pilot-study was to evaluate a new concept to improve and organize clinical practice in order to get insight on the different initiatives and gain knowledge to further developing. The objective of a pilot-study is often to assess randomization, recruitment strategies, and sample size calculation, or to test the acceptability of an intervention, the feasibility of a study protocol, or the selection of the most appropriate primary outcomes measure [33]. In addition, a pilot-study is not perceived as strong evidence [12, 13], and this pilot-study do not provide data on the effects of the new concept on nursing students clinical learning environment, feeling of belonging and well-being, relationship with healthcare staff, and facilitation of the transition from student to working life in comparison to traditional clinical practice. This is planned to be investigated in a forthcoming study. In addition, we wish to explore the experience of the new concept among healthcare staff at the departments and nurse educators from the nursing education.

We acknowledge the fact that the results are interpreted in the light of the limitations that might occur in connection with a single-center, pilot-study with a small sample-size. The results are therefore not generalizable and no general conclusion can be drawn from this study. However, we believe our study showing nursing students, clinical supervisors, and head nurses positive experiences regarding the new concept to be a useful contribution to the sparse knowledge on improving and organizing clinical practice of nursing students and may act as an inspiration when planning other intervention studies.

## Conclusion

In conclusion, this pilot-study found that affiliation to the same department during clinical practice together with participation in reflective supervision and a self-compassion course was experienced positive among nursing students, clinical supervisors, and head nurses. In addition, the new concept contributed to the experience of a safe learning environment, increased well-being among nursing students, strengthen relationship with the clinical supervisors and healthcare staff at the department, and prepared the nursing students to working life. Our results complement the suggestion that improved quality of clinical practice for nursing students is an effective strategy to establish a safe and supportive learning environment that contribute with satisfaction, successful experiences, and attraction of future nurses. Our results have important implication to strengthen the collaboration between clinical workplaces and nursing educations. However, further intervention studies are needed to compare the effect of the new concept with traditional clinical practice.

### Supplementary Information

**Supplementary Material 1.** 

**Supplementary Material 2.** 

## Data Availability

Data is provided within the manuscript and supplementary information files. Due to the small sample size, the dataset generated during this study is not available.

## Abbreviations

ECTS: European Credit Transfer System
GDPR: General Data Protection Regulation
