# Supplementary material for: Evaluation of a new concept to improve and organize clinical practice in nursing education: a pilot-study
Source: BMC Nurs. 2024 Mar 26;23:203. doi: 10.1186/s12912-024-01888-y (PMC10964657; doi:10.1186/s12912-024-01888-y)
Supplement: Supplementary file 1 — Supplementary Material 1. [file 12912_2024_1888_MOESM1_ESM.docx]

**Supplementary Tabel 1. Questionnaires**

Unless other stated, the questions were rated using a five point Likert-scale ranging from: to a very low degree, to a low degree, to some extent, to a high degree, and to a very high degree.

| **Questionnaires to nursing students** | |
| --- | --- |
| Themes |  |
| **Information and involvement during the new concept** |  |
| - Did you participate in the information meetings? (yes or no) |  |
| - Did you feel well informed prior clinical practice? |  |
| - Did you feel well informed during clinical practice? |  |
| - Did the online information forum keep you informed? |  |
| **Safety, well-being, and learning outcome** |  |
| - Did the new concept create a safe learning environment? |  |
| - Did you feel a part of the healthcare staff? |  |
| - Did you feel safe knowing you was affiliated to the same department? |  |
| **Transition from student life to working life** |  |
| - Did the new concept contribute to prepare you to become a nurse? |  |
| **Impact of reflective supervision and self-compassion course** |  |
| - Did you participate in the reflective supervision? (yes or no) |  |
| - Did reflective supervision improve your ability to apply theoretical knowledge to practice? |  |
| - Did reflective supervision bring new perspectives on clinical issues? |  |
| - Did reflective supervision bring new perspectives on development of quality? |  |
| - Did reflective supervision bring? new perspectives on interdisciplinary and cross-sectoral collaboration |  |
| - Did reflective supervision contribute to community of practice? |  |
| - Did the self-compassion course improve your well-being? |  |
| - Do you have any further comments you want to share about the new concept – please write here: |  |
|  | |
| **Questionnaires to clinical supervisors** | |
| Themes |  |
| **Information and involvement during the new concept** | |
| - Did you participated in at least one information meetings? (yes or no) |  |
| - Did you feel well informed about the new concept? |  |
| - Did the online information forum keep you informed? |  |
| - Did you feel involved in the new concept? |  |
| **Safety, well-being, and learning outcome** | |
| - Did the new concept improve learning for the nursing students? |  |
| - Did the new concept improve well-being for the nursing students? |  |
| - Did the new concept promote the well-being of the nursing students to be affiliated to the same department? |  |
| **Transition from student life to working life** | |
| - Did the new concept contribute to a higher degree of professional identity among the nursing students? |  |
| **Impact of reflective supervision and self-compassion course** | |
| - Did the reflective supervision improve the nursing students’ ability to apply theoretical knowledge to practice? |  |
| - Did you experience that the nursing students used elements from the self-compassion course? |  |
| - Did the self-compassion course contribute to the nursing students learning and well-being? |  |
| - Did the self-compassion course valuable in regards to your position as a clinical supervisor? |  |
| - Did you use compassion during your work as a clinical supervisor? |  |
| - Do you have any further comments you want to share about the new concept – please write here: |  |
|  | |
| **Questionnaires to head nurses** | |
| Themes |  |
| **Safety, well-being, and learning outcome** | |
| - Did the new concept improve learning for the nursing students? |  |
| - Did the new concept improve well-being for the nursing students? |  |
| **Transition from student life to working life** | |
| - Did the new concept contribute to higher degree of professional identity? |  |
| - Do you have any further comments you want to share about the new concept – please write here: |  |
