# Supplementary material for: Evaluation of a new concept to improve and organize clinical practice in nursing education: a pilot-study
Source: BMC Nurs. 2024 Mar 26;23:203. doi: 10.1186/s12912-024-01888-y (PMC10964657; doi:10.1186/s12912-024-01888-y)
Supplement: Supplementary file 2 — Supplementary Material 2. [file 12912_2024_1888_MOESM2_ESM.docx]

**Supplementary Tabel 2. Interview guides**

| **Nursing students and clinical supervisors** | |
| --- | --- |
| Introduction to the aim of the pilot-study |  |
| Description of the focus group interview |  |
|  |  |
| The new concept | How would you describe the new concept for clinical practice? |
|  |  |
|  | What was your experience with the new concept? |
|  |  |
|  | What do you think are good about the new concept? |
|  |  |
|  | What do you think could be improved? |
|  |  |
|  | What was your aspirations for the new concept? |
|  |  |
| Reflective supervision | What was your experience of reflective supervision? |
|  |  |
|  | What do you think was good about reflective supervision? |
|  |  |
|  | Anything that need to be changed? |
|  |  |
| Self-compassion course | What was your experience with the self-compassion course? |
|  |  |
|  | What do you think was good about the self-compassion course |
|  |  |
|  | Anything that need to be changed? |
|  |  |
| Ending of the focus group interviews |  |
| Any last comments |  |
